# Chromosome Mapping of Dragline Silk Genes in the Genomes of Widow Spiders (Araneae, Theridiidae)

**DOI:** 10.1371/journal.pone.0012804

**Published:** 2010-09-21

**Authors:** Yonghui Zhao, Nadia A. Ayoub, Cheryl Y. Hayashi

**Affiliations:** Department of Biology, University of California Riverside, Riverside, California, United States of America; American Museum of Natural History, United States of America

## Abstract

With its incredible strength and toughness, spider dragline silk is widely lauded for its impressive material properties. Dragline silk is composed of two structural proteins, MaSp1 and MaSp2, which are encoded by members of the spidroin gene family. While previous studies have characterized the genes that encode the constituent proteins of spider silks, nothing is known about the physical location of these genes. We determined karyotypes and sex chromosome organization for the widow spiders, *Latrodectus hesperus* and *L. geometricus* (Araneae, Theridiidae). We then used fluorescence *in situ* hybridization to map the genomic locations of the genes for the silk proteins that compose the remarkable spider dragline. These genes included three loci for the MaSp1 protein and the single locus for the MaSp2 protein. In addition, we mapped a *MaSp1* pseudogene. All the *MaSp1* gene copies and pseudogene localized to a single chromosomal region while *MaSp2* was located on a different chromosome of *L. hesperus*. Using probes derived from *L. hesperus*, we comparatively mapped all three *MaSp1* loci to a single region of a *L. geometricus* chromosome. As with *L. hesperus*, *MaSp2* was found on a separate *L. geometricus* chromosome, thus again unlinked to the *MaSp1* loci. These results indicate orthology of the corresponding chromosomal regions in the two widow genomes. Moreover, the occurrence of multiple *MaSp1* loci in a conserved gene cluster across species suggests that *MaSp1* proliferated by tandem duplication in a common ancestor of *L. geometricus* and *L. hesperus*. Unequal crossover events during recombination could have given rise to the gene copies and could also maintain sequence similarity among gene copies over time. Further comparative mapping with taxa of increasing divergence from *Latrodectus* will pinpoint when the *MaSp1* duplication events occurred and the phylogenetic distribution of silk gene linkage patterns.

## Introduction

With over 41,000 described species, spiders are one of the most diverse metazoan orders [1]. Numerous studies have been published on spider ecology, physiology, systematics, venom toxicology, silk biology, *etc.* [2]. Despite the prevalence of spiders and research on them, their genetic characterization remains extremely limited. To date, there are no sequenced spider genomes. Rather, genomic analyses are mostly limited to measures of genome size and cytogenetic studies [3], [4]. The cytogenetic work has been restricted to determinations of karyotype, diploid chromosome number, C-banding patterns, nucleolus organizer regions, and sex chromosomes [5] [6]–[7]. Although karyotypes of more than 500 species of spiders have been reported [4], no genes have been localized onto chromosomes.

Western black widow (*Latrodectus hesperus*) and brown widow (*L. geometricus*) spiders are in the cob-web weaver family, Theridiidae (Araneae). Previous studies on these two species have largely focused on systematics, venom pharmacology, and silk characterizations, such as in [8] [9] [10] [11] [12]–[13]. In terms of cytogenetics, there is one report on the diploid chromosome number of female brown widows [14], but no descriptions of the karyotypes of brown widow males or either sex of the Western black widow.

Spider dragline silk is renowned for its high tensile strength and toughness, with Western black widow draglines among the strongest on record [15], [16]. The two primary constituents of orb-weaver dragline silk are the proteins, major ampullate spidroins 1 and 2 (MaSp1 and MaSp2) [17]. Complementary DNA (cDNA) encoding MaSp1 and MaSp2 of the golden orb-weaver, *Nephila clavipes*, were the first published spider silk gene sequences [18], [19]. These breakthroughs helped lead to the characterization of MaSp1 and MaSp2 from other species. For brown widows, MaSp1 and MaSp2 were described from conceptual translations of cDNA and genomic DNA clones[10], [20]. MaSp1 and MaSp2 of Western black widows were also initially characterized by cDNAs [21], [22].

Silk protein transcripts are typically very long and highly repetitive [23]. Given these attributes and the technical limitations of cDNA synthesis and cloning, nearly all spider silk genes are known only from partial-length cDNAs or gene fragments. Through construction of a large-insert genomic DNA library for the Western black widow, the first full-length *MaSp1* and *MaSp2* genes were deciphered, along with substantial amounts of flanking regions [11]. More recently, it was reported that *MaSp1* is a multi-copy gene in several spider species [24] [25]-[26]. For widow spiders, three *MaSp1* loci were diagnosed in the *L. hesperus* and *L. geometricus* genomes, and a *MaSp1* pseudogene was also found in *L. hesperus*. A series of PCR amplifications using DNA extractions from individual spiders were performed to distinguish between gene copies (separate loci) and allelic variation [25]. There was no evidence for more than one *MaSp2* gene in *L. hesperus*.

The spatial relationships of the *MaSp1* multiple loci and *MaSp2* locus in *Latrodectus* genomes are unknown. Indeed, to our knowledge, no silk gene has ever been physically located within any spider genome. To map the locations of the major ampullate spidroin loci, we use fluorescence *in situ* hybridization (FISH). FISH is typically performed with probes and targets derived from the same species. However, it can also be used for comparative mapping of genomic fragments onto the chromosomes of closely related species, without additional cloning or DNA sequencing, as shown in [27], [28].

The construction of a large-insert genomic DNA library for *L. hesperus* provides a rich resource for FISH probes that can be used to map genes and trace genome evolution in widow spiders [11]. To enable such studies, here we present the karyotypes and chromosomal C-banding for *L. hesperus* and *L. geometricus*. Next, we map five fosmid clones of *L. hesperus* to specific chromosomes of *L. hesperus* with FISH. Each clone harbors one of three distinct *MaSp1* gene copies, a *MaSp1* pseudogene, or *MaSp2.* Furthermore, the three *Masp1* gene copies and *Masp2* are heterologously mapped onto the karyotype of *L. geometricus*. Through these efforts, we aim to test scenarios of how the spidroin gene family evolved. If the silk genes are dispersed among chromosomes, that may indicate the birth of new paralogs via retroposition, among other possibilities. However, if the silk genes are localized to a single region in the genome, that pattern would be consistent with gene duplication from unequal crossing over events [29].

## Results and Discussion

### Karyotypes of L. hesperus and L. geometricus

**Figure 1:**
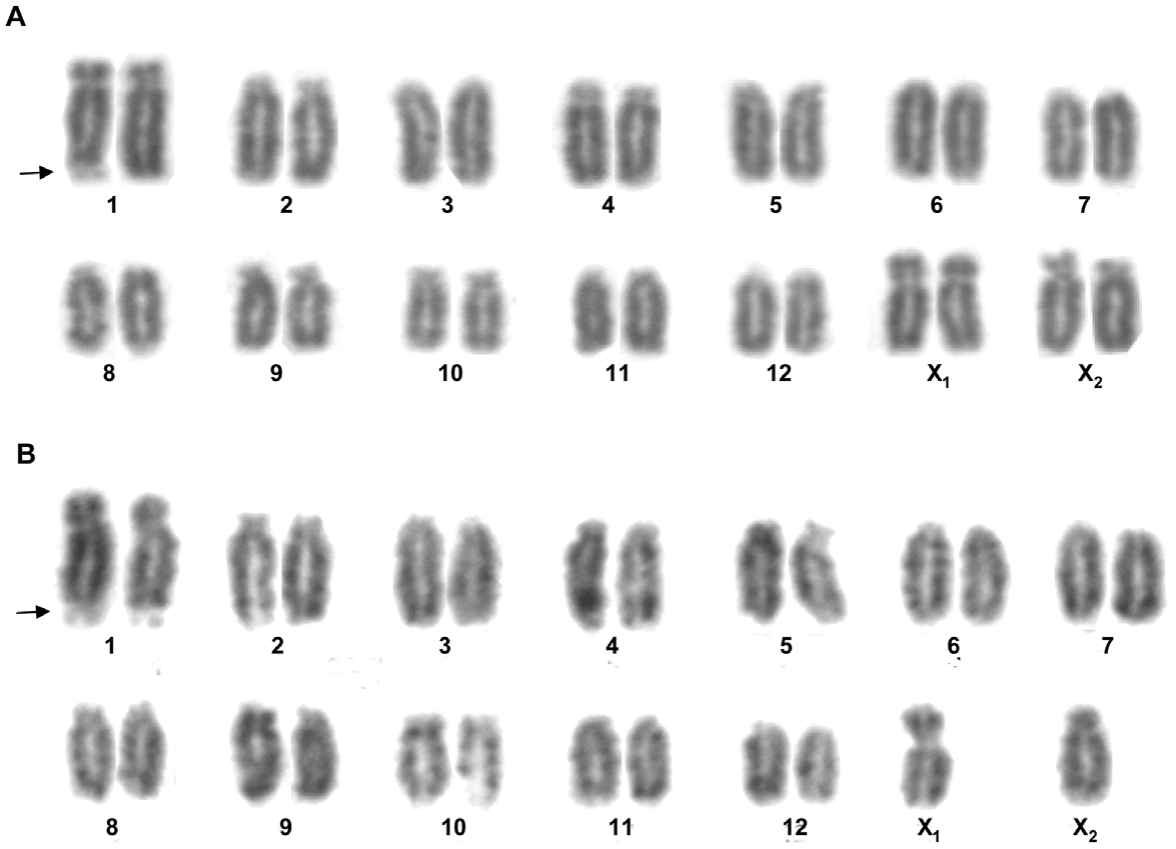
Karyotypes of *L. hesperus*. (A) Metaphase spread with 28 chromosomes (female). (B) Metaphase spread with 26 chromosomes (male). Autosomes are numbered and sex chromosomes designated as “X_1_” or “X_2_.” Arrows point to the terminal secondary constriction of the first autosome pair. Original metaphase spreads shown in Figures S1A and S1B.

We obtained over 100 well-defined metaphase spreads from developing *L. hesperus* (LH) eggs (20 eggs analyzed, 5–6 spreads per egg). From these visualizations, we determined that Western black widows have diploid chromosome numbers of 26 or 28 (Figures 1, S1A, S1B). These counts are identical to the 26/28 chromosome sets reported for both *L. indistinctus* and *L. curacaviensis* [14], [30]. Aside from a submetacentric chromosome that is present singly in the 26 chromosome sets and paired in the 28 chromosome sets, all *L. hesperus* chromosomes are telo/acrocentric.

The autosome pairs in the *L. hesperus* karyotypes are numbered according to descending size (Figure 1). The largest autosome pair of *L. hesperus* (LH-1) has a secondary constriction and a satellite chromosome at the end of the long arm (Figures 1, S1A, S1B). One or two additional, small, telocentric chromosomes are infrequently found in the metaphase spreads (Figure S2). These small elements could be supernumerary chromosomes (e.g., B-chromosomes), which are known from eukaryotes such as grasshoppers, wasps and plants [31]. Because supernumerary chromosomes are often not stably transmitted, this could explain the variable number (zero, one, or two) of these small elements among *L. hesperus* karyotypes. The significance of the satellite and supernumerary chromosomal elements, and their polymorphic distribution among *L. hesperus* individuals, is an area for future inquiry.

**Figure 2:**
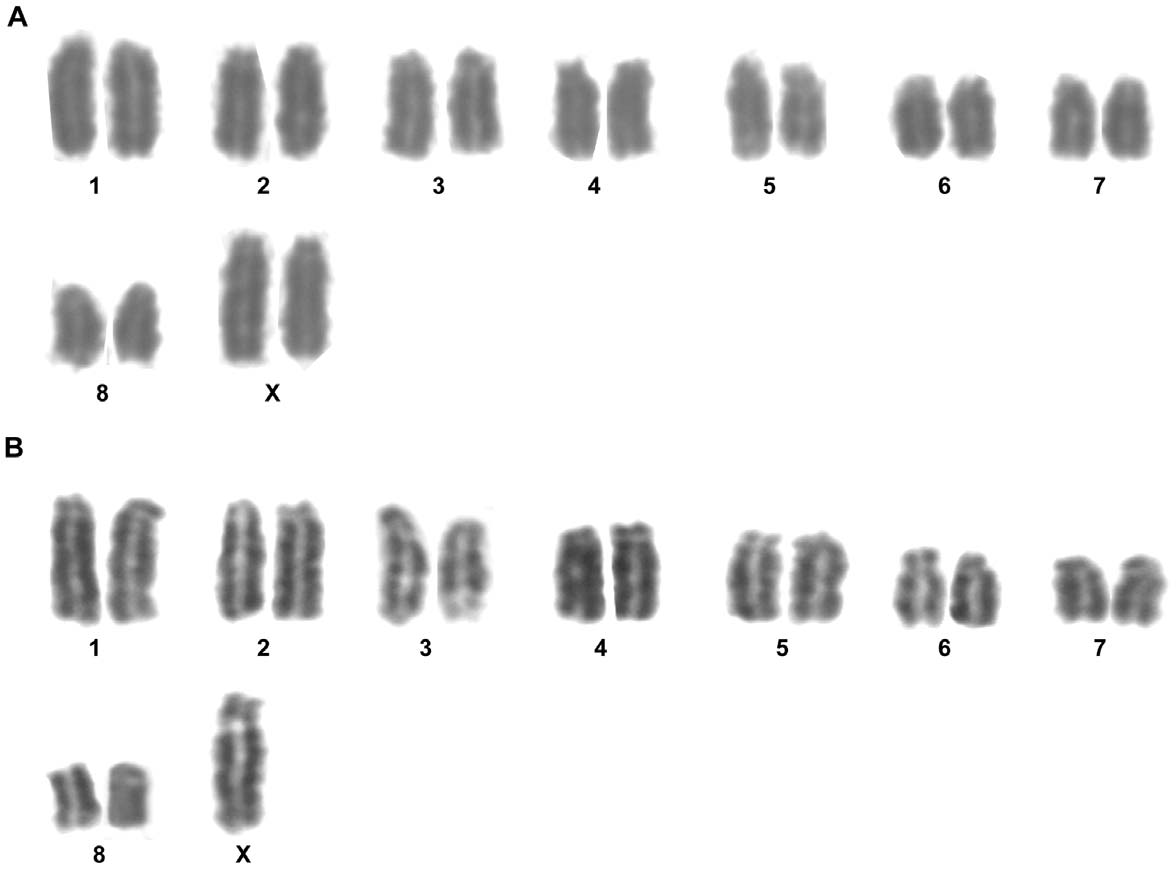
Karyotypes of *L. geometricus*. (A) Metaphase spread with 18 chromosomes (female). (B) Metaphase spread with 17 chromosomes (male). Autosomes are numbered and sex chromosomes designated by “X.” Original metaphase spreads shown in Figures S1C and S1D.

For *L. geometricus* (LG), ∼50 high-quality metaphase spreads were derived from 20 developing eggs. There were primarily two types of cells, those with a diploid chromosome number of 17 and those with 18 (Figures 2, S1C, S1D). All chromosomes were telo/acrocentric and no supernumerary chromosomes were observed. As with the *L. hesperus* karyotype, *L. geometricus* autosome pairs were numbered by descending size (Figure 2).

### Sex chromosome organization

In spiders, males and females of a species usually differ in the number of sex chromosomes they possess [6], [32]. Females typically have more copies of the sex chromosomes. The most common sex chromosome organization of spiders in the Entelegynae, which includes theridiids, is an X_1_X_2_0 system [7]. In this scheme, there are two pairs of X chromosomes in females (X_1_X_1_X_2_X_2_), while males have two unpaired X chromosomes (X_1_X_2_). Thus, the diploid chromosome number of female spiders is commonly higher than that of males. Various other X chromosome systems (e.g., X0, X_1_X_2_X_3_0, X_1_X_2_X_3_X_4_0), as well as some XY systems (e.g., X_1_X_2_X_3_Y), have also been reported in spiders [33]–[35].

For both *L. hesperus* and *L. geometricus*, developing eggs, not individuals of known sex, were used to obtain the metaphase spreads. Thus additional information is needed to associate each karyotype with its respective sex. Considering the *L. hesperus* chromosome morphology and diploid chromosome numbers of 26 and 28, we deduce that the sex chromosome organization of the Western black widow is X_1_X_2_ in males (2n = 26) and X_1_X_1_X_2_X_2_ in females (2n = 28). The X_1_ sex chromosome is identifiable by its unique, submetacentric appearance and its presence as either single or paired in the two types of metaphase chromosome spreads that we observed. The X_2_ sex chromosome is distinguishable by its short arm (p), which is longer than those of similar-sized acrocentric autosomes (Figure 1).

*L. geometricus* metaphase spreads have either 17 or 18 diploid chromosomes (Figure 2). The count of 18 chromosomes is consistent with the karyotype for a female *L. geometricus* that was previously described [14]. Hence, the sex chromosome organization of *L. geometricus* is X0 (an unpaired X) in males (2n = 17) and XX in females (2n = 18). The brown widow X chromosome is distinguishable as the largest chromosome in the genome (Figure 2).

Further investigation is needed to determine the relationship between the two X chromosomes of *L. hesperus* and the single X chromosome of *L. geometricus*. Possible evolutionary scenarios include the origin of the *L. geometricus* X from fusion of two ancestral sex chromosomes, or the derivation of the two *L. hesperus* X chromosomes from fission of a single ancestral sex chromosome. Insight can be gained by considering the karyotypes of closely related species. The subfamily Latrodectinae is composed of three genera, *Latrodectus*, *Steatoda*, and *Crustulina* [36]. Karyotypes with 22 and 24 chromosomes have been published for *Steatoda bipunctata, S. grossa, S. triangulosa*, and *Crustulina guttata*, indicating that these species all have two sex chromosomes [30], [37]. Considering that *L. hesperus*, *L. indistinctus*, and *L. curacaviensis* also have two sex chromosomes, this is most likely the ancestral condition in latrodectine spiders [14], [30]. If so, then the single X chromosome in *L. geometricus* arose through chromosomal fusion. When molecular markers are developed for the *Latrodectus* X chromosomes, this hypothesis can be tested.

### C-banding of L. hesperus and L. geometricus

**Figure 3:**
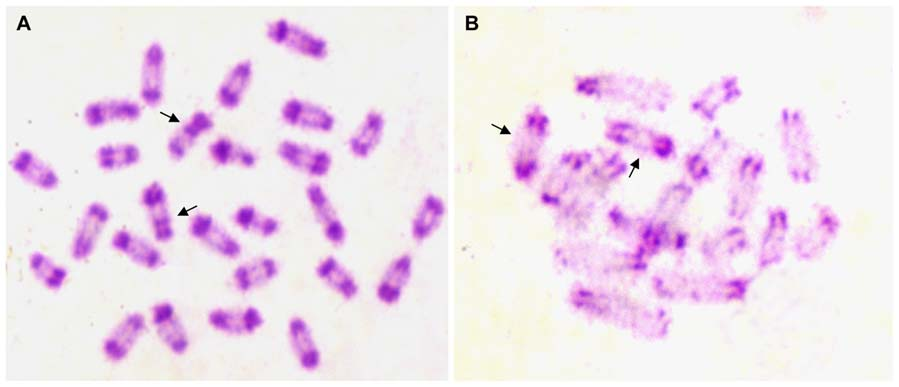
C-banding of chromosomes from male *Latrodectus*. (A) *L. hesperus* with arrows pointing to sex chromosomes X_1_ and X_2_, and (B) *L. geometricus* with arrows pointing to LG-4.

C-bands are regions of chromosomes that are formed by constitutive heterochromatin. They stain darkly due to the dense packing of chromatin. For many organisms, C-bands can serve as chromosomal markers because the distribution of heterochromatin varies among chromosomes within a genome and between the genomes of different species. In *L. hesperus*, we found that most of the C-bands are located in the telomeric and centromeric areas of each chromosome (Figure 3A). The chromosomes have similar banding patterns, except that the sex chromosomes have extra C-bands on their long arms, toward the centromeres (arrows in Figure 3A). In *L. geometricus*, the majority of C-bands are in the centromeric region of chromosomes, and LG-4 is distinctive in having more heterochromatin than any of the other chromosomes (Figure 3B).

The C-banding of *L. hesperus* is extensive, indicating that the ratio of constitutive heterochromatin to euchromatin is high in the *L. hesperus* genome. By contrast, there is much less C-banding, and hence relatively lower amounts of constitutive heterochromatin on *L. geometricus* chromosomes (Figure 3). The greater proportion of C-banding in *L. hesperus* compared to *L. geometricus* cannot be easily explained by genome size since *L. hesperus* has the smaller genome (1.29 pg for *L. hesperus* vs. 1.54 pg for *L. geometricus*) [38]. It is currently unknown whether there is functional significance to the variation in constitutive heterochromatin in *Latrodectus*, as has been reported in other eukaryotes [39].

### Mapping of *MaSp1* and *MaSp2* onto *Latrodectus* chromosomes by FISH

**Figure 4:**
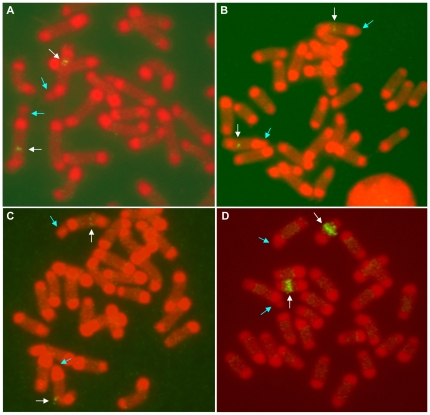
FISH mapping of *L. hesperus* chromosomes with probes. (A) *MaSp1* locus 1, (B) *MaSp1* locus 2, (C) *MaSp1* locus 3, and (D) *MaSp2.* White arrows point to hybridization signals, blue arrows to the secondary constriction of LH-1. Further details about probes and signal locations are in Table 1.

Clones from an *L. hesperus* fosmid library were successfully hybridized to chromosomes of *L. hesperus* and *L. geometricus*. The fosmid clones contained inserts of ∼40 kb of genomic DNA. Four fosmid clones, each of which contained one of three loci of *MaSp1* or the *MaSp1* pseudogene, were mapped to the same general region, the long arm (q) of LH-1 (LH-1q; Figures 4A–4C, S3). Further analysis showed that these clones are located in the same specific region, as determined by measuring the Fractional Length from the p terminus (FLpter) of their hybridization signals (Table 1). A fifth fosmid clone that contained *MaSp2* was mapped onto LH-8q (Figure 4D).

**Table 1:** Genomic location (chromosome number-arm) and FLpter values of *MaSp1* gene copies, *MaSp1* pseudogene, or *MaSp2* in *L. hesperus* (LH) and *L. geometricus* (LG).

Fosmid probe	Genbank Accession	LH location	LH Flpter	LG location	LG FLpter
*MaSp1* locus 1	EF595246	LH-1q	0.43±0.03	LG-4q	0.36±0.03
*MaSp1* locus 2	EU177653	LH-1q	0.44±0.03	LG-4q	0.34±0.03
*MaSp1* locus 3	EU177650	LH-1q	0.45±0.03	LG-4q	0.35±0.03
*MaSp1* pseudogene	EU177647	LH-1q	0.46±0.03	n/a	n/a
*MaSp2*	EF595245	LH-8q	0.44±0.05	LG-2q	0.72±0.05

Chromosomes numbered as in Figure 1. Each FLpter value is based on 8–15 metaphase spreads. In calculating the FLpter values on LH-1q, the relative lengths of secondary constrictions were not included because they were not visible in all metaphase spreads.

**Figure 5:**
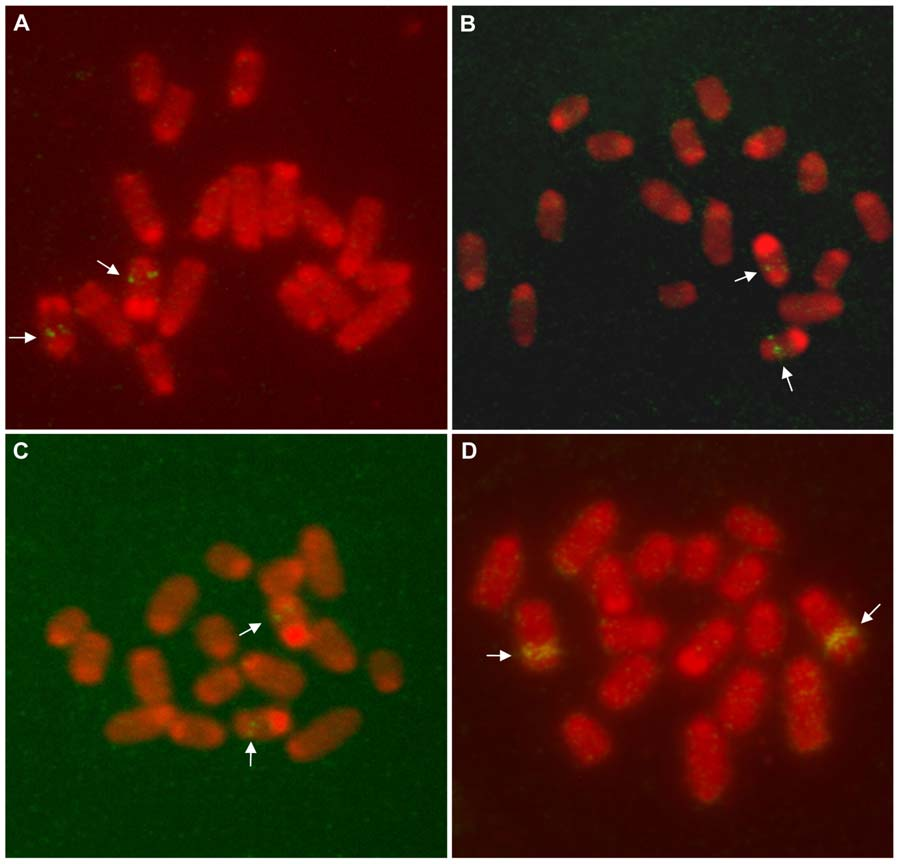
FISH comparative mapping of *L. geometricus* chromosomes with probes. (A) *MaSp1* locus 1, (B) *MaSp1* locus 2, (C) *MaSp1* locus 3, and (D) *MaSp2.* Arrows point to hybridization signals. Further details about probes and signal locations are in Table 1.

The three *L. hesperus* fosmid clones with *MaSp1* gene copies were successfully mapped onto *L. geometricus* chromosomes. All three gene copies hybridized to the long arm of the fourth largest autosome pair of *L. geometricus* (LG-4q; Figure 5A–5C). FLpter values indicate that the three loci are not just on the same arm of the same chromosome, but they are in the identical chromosomal region (Table 1). The *L. hesperus* fosmid clone with *MaSp2* was comparatively mapped to the long arm of the second-largest autosome pair of *L. geometricus* (LG-2q; Figure 5D).

The relative sizes of the *MaSp1* and *MaSp2* containing chromosomes vary between *Latrodectus* species. In *L. hesperus*, the *MaSp1* loci are on LH-1, the largest chromosome, while *MaSp2* is on LH-8, a considerably smaller chromosome. By contrast, in *L. geometricus*, *MaSp2* is on a larger chromosome (LG-2) than the *MaSp1* loci (LG-4). Furthermore, as indicated by FLpter values, *L. hesperus MaSp2* is near the middle of the chromosome long arm, while in *L. geometricus*, the gene is close to the telomere (Table 1). Thus, the FLpter values and morphometry of the *MaSp1* and *MaSp2* bearing chromosomes are signposts of the genomic rearrangements that have occurred since *L. geometricus* and *L. hesperus* diverged from their most recent common ancestor.

### Evolution of *Latrodectus* silk genes and genomic regions

Based on analyses of mitochondrial cytochrome c oxidase subunit 1, the genus *Latrodectus* is divided into two monophyletic groups, the *geometricus* clade and the *mactans* clade [8]. The *geometricus* clade is named after its member, *L. geometricus*, and the *mactans* clade includes *L. hesperus*. Thus, *L. geometricus* and *L. hesperus* span the maximum phylogenetic divergence in *Latrodectus*. Differences in number of autosome pairs, sex chromosome systems, and pattern of constitutive hetrochromatin are further evidence of the marked genomic changes between these species (Figures 1 – 3). Yet, by comparative mapping of *MaSp1* and *MaSp2* genes with FISH, we are able to predict orthology of genomic regions on LH-1q with LG-4q, and LH-8q with LG-2q (Table 1).

The mapping of multiple *MaSp1* genes to single chromosomal regions in *L. hesperus* and *L. geometricus* (Figures 4 and 5) implies that in widow genomes, the *MaSp1* loci are in a gene cluster. Based on the resolution of FISH with metaphase chromosomes, we deduce that this gene cluster is within a 2–3 Mb region [40]. Neighboring of multicopy genes has also been observed in other organisms, such as *DAZ* fertility factor genes on the human Y chromosome, patatin genes in potato, and histone genes in grasshopper [41] [42]–[43]. The *MaSp1* gene cluster contains a minimum of three copies and it is possible that there are more copies that have yet to be characterized. However, if there are additional copies, there is no evidence that they are located outside of the gene cluster. Given that the full-length *MaSp1* gene copy (locus 1) is a ∼10 kb single exon gene, cross-hybridization could occur among gene copies due to the substantial amounts of sequence conservation observed among the three known loci [11], [25]. Yet, FISH did not reveal signal outside of the gene cluster region, which would have indicated dispersion of *MaSp1* loci in *L. hesperus* or *L. geometricus* (Figures 4A–4C, 5A–5C).

The presence of *MaSp1* gene clusters in both *Latrodectus* species, suggests that *MaSp1* gene duplications predate their speciation. If the multiple *MaSp1* loci in *Nephila* (Nephilidae) [26] and *Euprosthenops* (Pisauridae) [24] are also similarly localized in their respective genomes, then the *MaSp1* gene cluster could be extremely ancient. Based on the estimated divergence time of these spider lineages, the gene cluster could have persisted for over 200 million years [44]. Alternatively, *MaSp1* gene clusters could have multiple origins. To distinguish between the scenarios of single vs. convergent evolution of gene clusters, the genomic distribution of *MaSp1* genes and adjacent chromosomal regions need to be mapped across spider families. Regardless of the history of the *MaSp1* multiple loci, there appears to be selection for the maintenance of the gene copies. Possible functional significance could be to increase gene dosage or as part of tissue-specific, developmental-stage, or other condition-specific differential gene expression [25].

The localization of *Latrodectus MaSp1* loci to a single chromosomal region is also consistent with the spawning of gene copies by tandem duplication. Segmental duplication via unequal recombination of sister chromatids is one mechanism that can generate new paralogs [29], [45]. Fibroin genes in particular are especially prone to unequal recombination because they are largely composed of repetitive sequences [46]. For example, the aciniform spidroin gene (*AcSp1*) from the banded garden spider, *Argiope trifasciata*, is built from a tandem array of 600 bp repeat units, some of which share an extraordinary 100% pairwise nucleotide identity [47]. Remarkable sequence conservation has also been noted in other members of the spidroin gene family, including *MaSp1* and *MaSp2* from widow spiders [11], [22], [48], [49]. Specifically, in the *L. hesperus MaSp1* locus 1, there are 20 tandem-arrayed iterations of a repeat that average a scant 2.5% pairwise sequence divergence over 348 nucleotides [11]. The unusual repetitive design of silk genes provides ample opportunities for misalignment of chromosomes during meiosis and unequal crossing over. The genetic shuffling caused by these events results in duplication or deletion not only of intragenic repeat units (e.g., allelic length variants; [50]), but also of entire gene copies (e.g., tandem duplicates). The genomic localization of multiple *MaSp1* loci in *Latrodectus* is consistent with the persistence of tandem duplicates over evolutionary time.

Tandem duplication of *MaSp1* results in tandem arrayed gene copies. This physical arrangement is a higher level of repetitiveness beyond the ensemble repeats and subrepeats previously described for spider silks. Sequence conservation among tandem repeat units within a gene can occur by concerted evolution that homogenizes the repeats throughout an array. Similarly, physical proximity on a chromosome can explain much of the sequence conservation noted for *Latrodectus MaSp1* loci [25] through concerted evolution of genes, such as observed for arrays of rRNA genes [51].

The location of *MaSp2* on a different chromosome from the *MaSp1* loci in *Latrodectus* species is also informative about the evolutionary dynamics of the spidroin gene family (Table 1). Phylogenetic analyses of *MaSp1* and *MaSp2* indicate that they form a clade to the exclusion of other spidroin gene family members [17], [20], [22]. Furthermore, *Latrodectus MaSp2* appears to have a *MaSp1* ancestor, meaning that *MaSp2* is a divergent *MaSp1* paralog [25]. This inference is in agreement with the demonstration from FISH mapping that *MaSp2* is unlinked to *MaSp1*. Accordingly, *MaSp2* likely originated from a *MaSp1* gene copy either via retroposition of a *MaSp1* transcript (hence, *MaSp2*′s disparate location), or translocation to a different chromosome. Once unlinked from the other *MaSp1* paralogs, the repetitive region of *MaSp2* dramatically diverged in sequence from *MaSp1*. Analyses to detect past recombination events, however, reveal that there has been recombination between the amino- and carboxyl-termini encoding regions of *Latrodectus MaSp1* loci and *MaSp2*. Yet, this recombination rate is less frequent than the homogenization among *MaSp1* loci [25].

In summary, the karyotyping of the brown widow and Western black widow revealed their autosome and sex chromosome organization (Figures 1 and 2). Characterization of these karyotypes enabled the first comparative mapping of spider genomes. FISH was used to locate the positions of multiple *MaSp1* gene copies and the *MaSp2* gene in *Latrodectus* species (Figures 4, 5, S3). Given the substantial phylogenetic divergence, differences in genomic organization, and near absence of genomic sequence data for these species, our results attest to the power of FISH for gaining insight into the molecular evolution of understudied organisms.

The comparative genomic hybridization results shed light on the evolutionary dynamics of the *MaSp1* and *MaSp2* genes that underlie the structural proteins of one of nature's most renowned materials, the spider dragline. *MaSp1* loci, including a pseudogene, are clustered (Figures 4, 5, S3; Table 1), consistent with the origin of paralogs through segmental duplication events and homogenization via concerted evolution [29], [45]. *MaSp2*, which is closely related to *MaSp1* [25], is unlinked from the *MaSp1* array. This physical separation is consistent with the substantial differences that have accumulated between MaSp1 and MaSp2. Indeed, MaSp2 has distinctive attributes, such as prevalent proline residues, that contribute extensibility and toughness to spider draglines [23]. Thus, the mapping of silk genes on chromosomes has implications for understanding the genomic environments in which the members of the spider silk gene family evolved to produce a diversity of high-performance biomaterials.

## Materials and Methods

### Preparation of chromosome spreads

Mature female *Latrodectus hesperus* (Western black widow) and *Latrodectus geometricus* (brown widow) were captured in Riverside and San Diego, California, respectively. Females were maintained in the lab and observed daily for the production of egg cases. Eggs were allowed to develop for 8–10 days and then were collected for chromosomes. For karyotyping, one egg was used per chromosome preparation. For FISH, multiple eggs were combined per preparation. Standard cytogenetic techniques were used to prepare and stain chromosome spreads [37], [52].

### Preparation of FISH probes

We cultured five fosmid clones that were isolated from a previously constructed *L. hesperus* genomic library [11]. The five clones harbored one of three *MaSp1* loci, a *MaSp1* pseudogene, or a *MaSp2* gene. Fosmid DNA was labeled with digoxigenin (Roche Applied Science) then co-precipitated with a 50-fold excess of salmon sperm DNA and sonicated *L. hesperus* genomic DNA. Immediately prior to hybridization, the precipitate was dissolved in hybridization solution (10% dextran sulfate sodium salt, 25% formamide, 2×SSC), denatured at 75°C, and put on ice.

### Fluorescence *in situ* hybridization

FISH was performed following previously described methods [53]. Briefly, slides of chromosome preparations were incubated at 60°C for 2 h, denatured at 72°C for 3 min in 70% formamide/2×SSC, and dehydrated at −20°C in an ethanol series. The denatured FISH probes were then added to the slides and held in place by cover glass. All five probes described above were used individually with *L. hesperus* chromosome preparations. Four of the probes (the functional genes) were used for heterologous FISH of *L. geometricus* chromosomes. The pseudogene containing probe was not used because it had only ∼500 bp of sequence identifiable as *L. hesperus MaSp1* and an orthologous region was not known in *L. geometricus*. Hybridizations were performed for 17 h at 37°C. After washing, hybridization signals were detected with mouse monoclonal anti-digoxigenin FITC antibodies that were amplified with rabbit anti-mouse IgG FITC and goat anti-rabbit IgG FITC antibodies (Sigma-Aldrich). Chromosomes were counterstained with propidium iodide. Images were captured with a Zeiss epi-fluorescence microscope equipped with a CCD camera.

## Supporting Information

Figure S1 Metaphase spreads used to derive the karyotypes shown in Figures 1 and 2. *L. hesperus* with (A) 28 chromosomes or (B) 26 chromosomes. Arrows point to second constriction of LH-1. *L. geometricus* with (C) 18 chromosomes, or (D) 17 chromosomes.
(1.24 MB TIF)

Figure S2 Metaphase spread from *L. hesperus* with arrows pointing to two supernumerary chromosomes.
(0.76 MB TIF)

Figure S3 FISH mapping of the MaSp1 pseudogene probe onto *L. hesperus* chromosomes. White arrows point to hybridization signals, blue arrows to the second constriction of LH-1. Further details about probe and signal location are in Table 1.
(0.88 MB TIF)
