# Supplementary figures and images for: Chromosome Mapping of Dragline Silk Genes in the Genomes of Widow Spiders (Araneae, Theridiidae)
Source: PLoS One. 2010 Sep 21;5(9):e12804. doi: 10.1371/journal.pone.0012804 (PMC2943472; doi:10.1371/journal.pone.0012804)

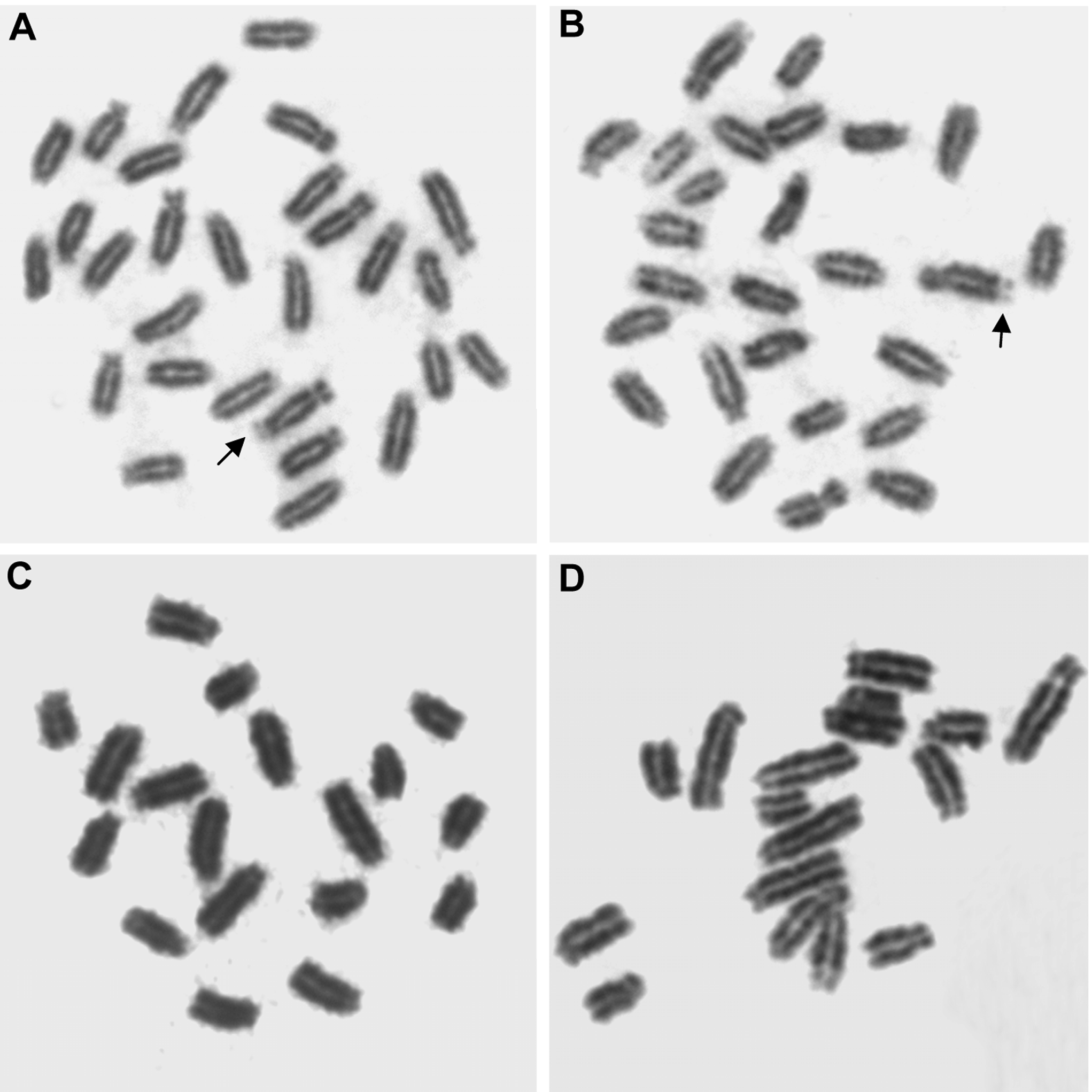

Supplement: Figure S1 — Metaphase spreads used to derive the karyotypes shown in Figures 1 and 2. L. hesperus with (A) 28 chromosomes or (B) 26 chromosomes. Arrows point to second constriction of LH-1. L. geometricus with (C) 18 chromosomes, or (D) 17 chromosomes. (1.24 MB TIF) [file pone.0012804.s001.tif]

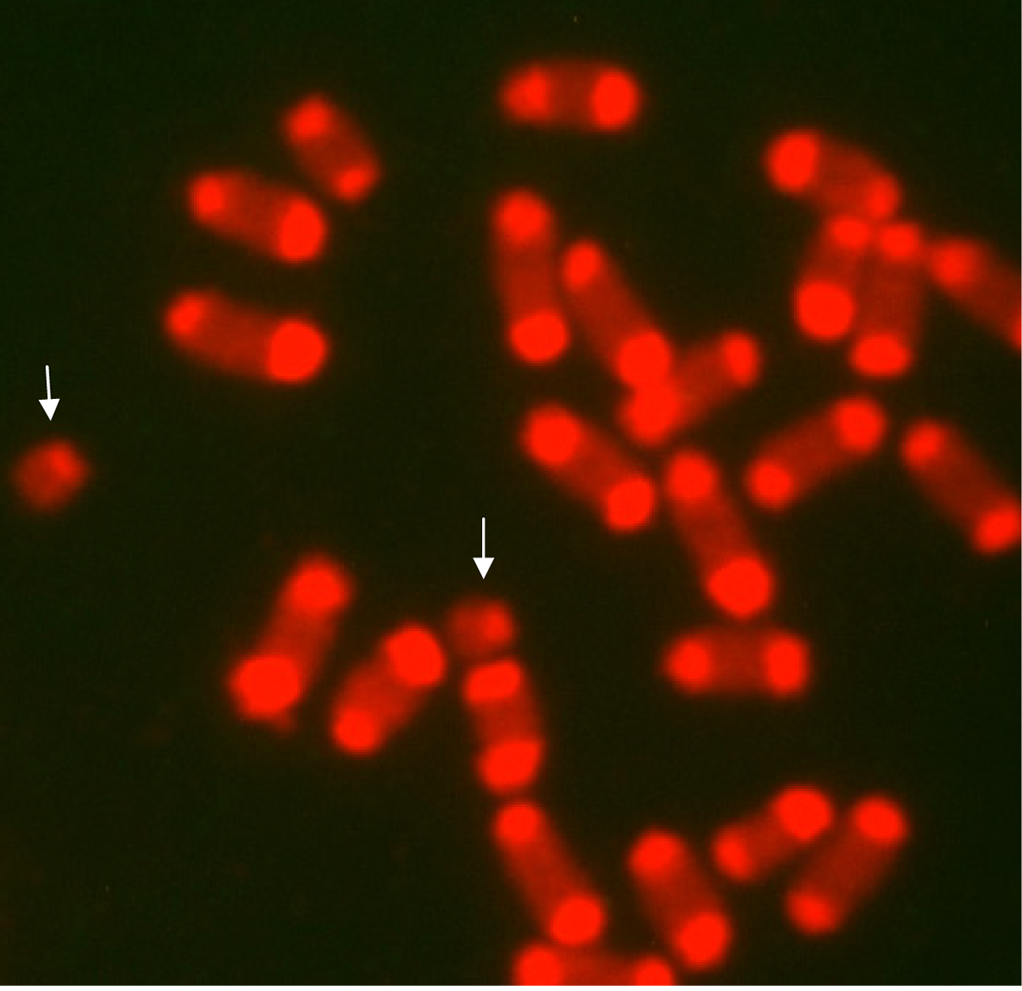

Supplement: Figure S2 — Metaphase spread from L. hesperus with arrows pointing to two supernumerary chromosomes. (0.76 MB TIF) [file pone.0012804.s002.tif]

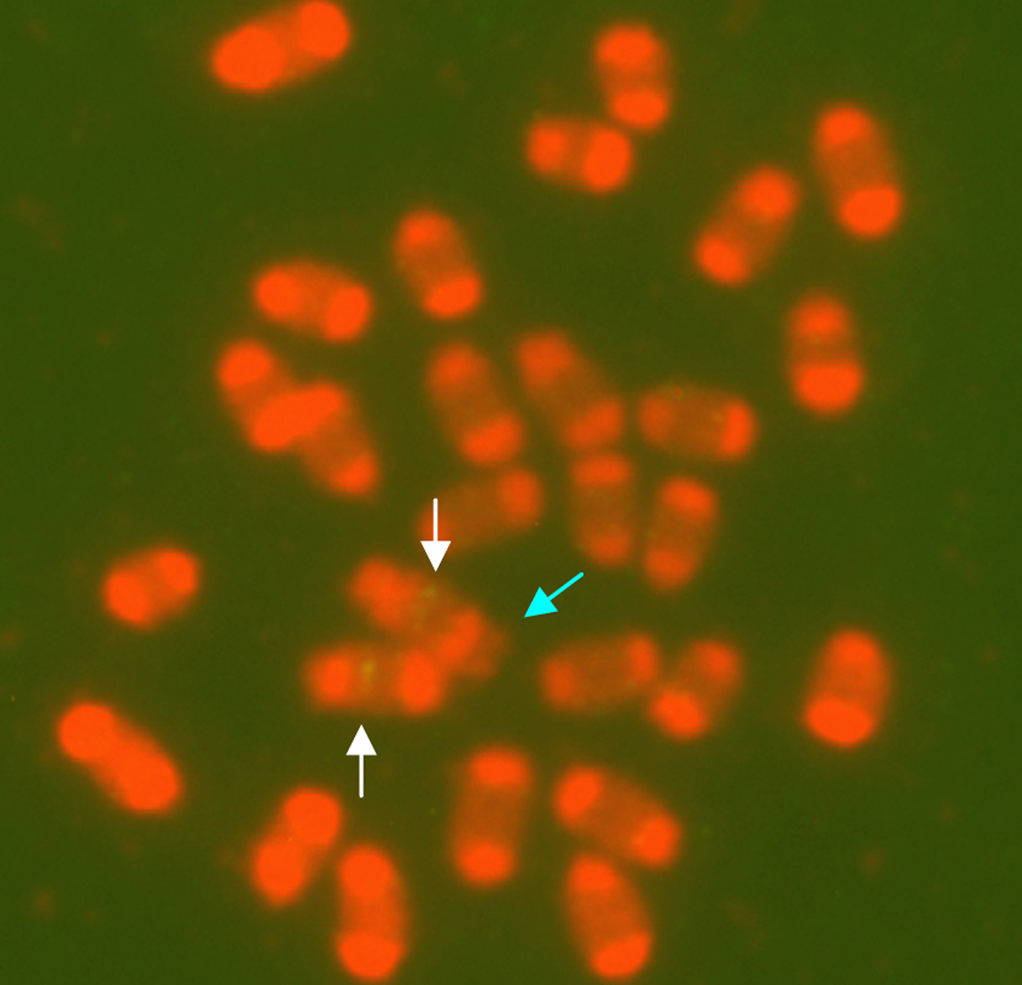

Supplement: Figure S3 — FISH mapping of the MaSp1 pseudogene probe onto L. hesperus chromosomes. White arrows point to hybridization signals, blue arrows to the second constriction of LH-1. Further details about probe and signal location are in Table 1. (0.88 MB TIF) [file pone.0012804.s003.tif]
